# Profile Change Law of Clad Rebars and the Formation Mechanism of Composite Interfaces during Hot Rolling

**DOI:** 10.3390/ma15217735

**Published:** 2022-11-03

**Authors:** Zhen Li, Xuehai Qian, Yong Xiang, Lei Zeng, Zecheng Zhuang, Jianping Tan

**Affiliations:** 1 School of Mechanical and Electrical Engineering, Central South University, Changsha 410083, China; 2 Hunan Provincial Engineering Research Centre for Laminated Metal Composites, Changsha 410083, China; 3 Technology Centre, Guangxi Liuzhou Iron and Steel Group Ltd., Liuzhou 545002, China; 4 Hunan Santai New Materials Ltd., Loudi 417000, China

**Keywords:** metal rheology law, composite interface generation mechanism, clad rebars, recrystallized grain, bond strength

## Abstract

Rough- and intermediate-rolled composite billets and finished clad rebars were cut using flying shears. The law of metal rheology and the mechanism of composite interface generation during clad rebar formation were then investigated using metallographic microscopy, electron backscatter diffraction, and scanning electron microscopy. The radial deformation trend of the clad rebars was greater than that of HRB400 rebars and “ears” were more likely to appear during the rolling process. The widths of the decarburization and composite zones and diffusion distances of each element decreased as the cumulative reduction rate increased. Furthermore, as deformation increased, the number of oxides on the composite interface significantly decreased, the proportion of recrystallized grains increased, and the grains became more refined. These changes led to increases in the bond and tensile strengths of the composite interface. According to the research above, the pass filling degree should be within 0.85–0.9 and the cumulative reduction rate should be over 80% when rolling clad rebars.

## 1. Introduction

According to statistics, the global economic loss caused by the corrosion of reinforced concrete exceeds USD 150 billion annually [1,2,3]. To retard the corrosion rate of reinforced concrete, researchers have proposed two methods: inhibit corrosion by adding a rust inhibitor to the concrete, which would increase both the quality and cost of the concrete [4,5], and prolong the corrosion period by using corrosion-resistant steel or cathodic protection methods [6,7].

At present, corrosion-resistant steel bars in the market are divided into epoxy-reinforced, galvanized, and stainless steel-reinforced bars. In engineering applications, epoxy reinforcements are prone to coating damage and corrosion failure [8]. Galvanized reinforcements have poor adhesion to concrete [9]. Stainless steel reinforcements are very costly to produce; thus, they are not widely used in the industry [10,11].

Clad rebars are a new type of composite reinforcement that use high-strength low-alloy steel as the base material, to which a cladding layer of austenitic or duplex stainless steel is added. The corrosion resistance of clad rebars is close to that of stainless steel bars but they are far less expensive. Thus, clad rebars have received much attention in recent years. Zhang et al. [12] proposed a process to prepare clad rebars by remelting carbon steel chips. First, the carbon steel chips are cleaned to remove oil and rust from their surface, after which they are filled into stainless steel tubes, compressed, and fused using a push rod. The resulting billets are then hot-rolled using continuous hot-rolling technology. The US company STELAX production process is similar to that of Zhang et al. in that the carbon steel chips are recycled, the difference is that The US company STELAX technique first compresses the carbon steel chips into rods and then rolls them into shape [13]. Sawicki et al. successfully prepared clad rebars using an explosive composite heating rolling process; here, carbon steel rods are loaded into stainless steel tubes by a clearance fit. Explosives are then used to create a shock wave that forms a preliminary mechanical bond between the carbon steel bar and stainless steel. Finally, the carbon steel bar/stainless steel tube is hot-rolled using a 150 mill with a flat elliptical–cylindrical hole pattern [14]. Feng et al. simulated the rolling process of clad rebars in the laboratory using a thermal simulator and a specific fixture. When the deformation was increased from 50% to 70%, all of the properties of the bars were improved [15]. Liu et al. prepared 9Cr18MoV/Cr13 clad rebars using a liquid–solid continuous casting method. First, the carbon steel is heated and liquefied using an induction heating furnace, after which the liquefied carbon steel is poured into stainless steel tubes, air-cooled to room temperature, and then rolled and formed by hot-rolling. The group also tested the tensile properties of the rolled clad rebars and found that clad rebars rolled using the above process had a yield strength of 433 MPa, which far exceeds the specified value of 400 MPa [16]. Gao et al. defined the steel composite interface by secondary development. First, the steel composite interface was defined as a friction constraint. During the simulation, the composite interface automatically became a co-nodal constraint when the stress of the composite interface exceeded the yield strength of the inner and outer materials [17]. Hua et al. used an electrochemical workstation to simulate the corrosion process of clad rebars. As the corrosion time increased, extensive pitting appeared on the surface of the clad rebars and the mechanical properties of the samples sharply decreased [18]. Xie et al. prepared Q195/304 clad rebars using a drawing and heating rolling process. First, the ground Q195 steel bar was snapped into a 304 stainless steel tube, after which the composite billet was drawn using a drawing machine to eliminate the gap between the bar and the stainless steel tube. A welded seal was then applied to the end face of the composite billet, and the sample was hot-rolled [19].

Owing to significant property differences between the base and cladding materials, inconsistent deformation can easily occur in clad rebars during the rolling process, leading to the surface leakage of the rebar and poor metallurgical bonding of the composite interface [20,21]. However, few scholars have studied the profile change law of clad rebars. Therefore, in this study, rough- and intermediate-rolled composite billets and finished clad rebars are cut using flying shears. The properties of these specimens are then investigated to determine the profile change law of clad rebars and the formation mechanism of composite interfaces during hot rolling. The findings could provide theoretical guidance for rolling process optimization.

## 2. Materials and Methods

### 2.1. Inner and Outer Layer Materials

The 20MnSiV and 316L stainless steels were selected as the base and cladding of the bars, respectively. Table 1 shows the chemical compositions of these steels.

### 2.2. Rolling Process

**Figure 1:**
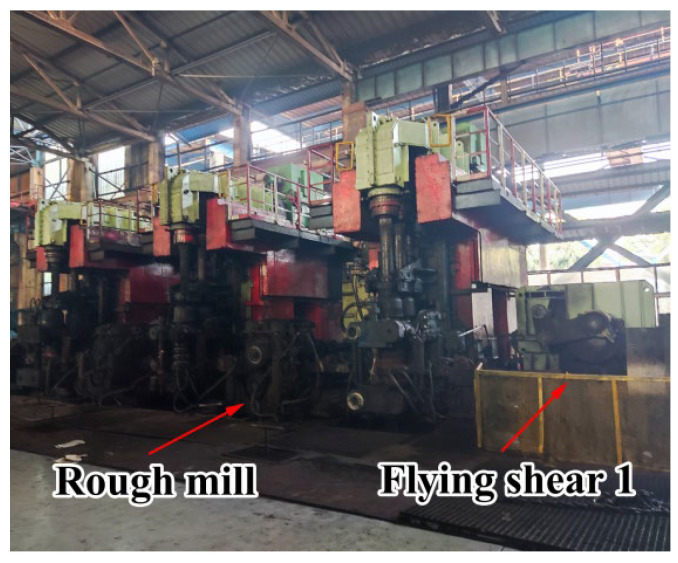
Rough-rolling mill with the first flying shear arrangement.

**Figure 2:**
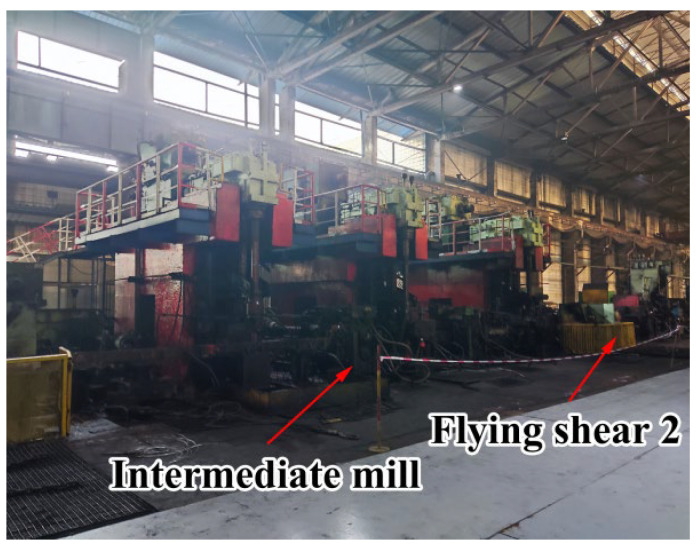
Intermediate-rolling mill with the second flying shear arrangement.

Rolling was carried out in Liuzhou Iron and Steel Co., Guangxi Province. The diameter of the composite billet was 159 mm, the thickness of the stainless steel pipe was 6 mm, and the diameter of the finished bar was 28 mm. The complete rolling line included 14 rolling mills and 2 flying shears. The rolling steps of the clad rebar are shown below. First, the composite billet was placed in a walking beam furnace for heating at 1250 °C for 3 h. After heating, the clad rebar was rolled using a rolling mill and air-cooled to room temperature. It is worth noting that the cooling water was closed during the whole rolling process for safeguarding the full recrystallization of clad rebar. Figure 1 shows a schematic of the rough-rolling unit and the first flying shear arrangement, while Figure 2 shows a schematic of the intermediate-rolling unit and the second flying shear arrangement. Table 2 shows the rolling parameters of the clad rebar.

**Figure 3:**
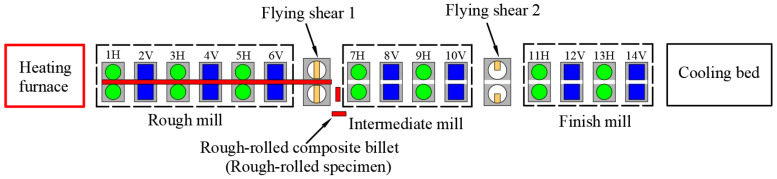
Schematic of the rough-rolling and specimen-cutting process.

The rough-rolled composite billets were prepared as follows. When the composite billet “head” reached the first flying shear position, a command was issued through the central control console and the flying shear quickly cut the billet “head”. A total of two specimens, each approximately 1 m long, were cut. Figure 3 shows a schematic of the rough-rolling and specimen-cutting process.

**Figure 4:**
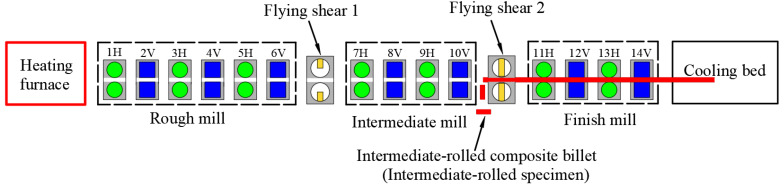
Schematic of the intermediate-rolling and specimen-cutting process.

Figure 4 shows a schematic of the intermediate-rolling and specimen-cutting process. The intermediate-rolled composite billets were prepared as follows. When the composite billet “tail” reached the position of the second flying shear, a command was issued through the central console and the flying shear quickly cut the billet “tail”. A total of two specimens, each approximately 1.5 m long, were cut.

**Figure 5:**
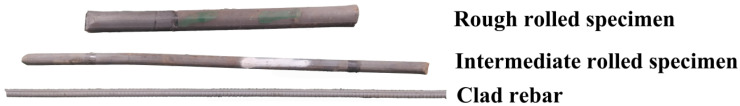
Surface morphology of the rough- and intermediate-rolled specimens and clad rebar.

The rough- and intermediate-rolled specimens were air-cooled to room temperature, numbered, and packed. Figure 5 shows the surface morphology of the rough- and intermediate-rolled specimens and clad rebar. According to the hole pattern provided by Liuzhou Iron and Steel Co., the cumulative reduction rates corresponding to the rough- and intermediate-rolled specimens were 47.5% and 73.3%, respectively; moreover, the cumulative reduction rate corresponding to the finished clad rebar was 82.3%.

### 2.3. Detection Methods

The machine vision method was employed to determine the stainless steel percentage of each specimen (i.e., rough- and intermediate-rolled specimens and clad rebar) as follows. First, a 33UX287 industrial camera (The Imaging Source, New Taipei City, Taiwan, China) was used to image the end-face of the specimens. The images obtained were then subjected to grayscale processing, noise reduction, adaptive thresholding, canny edge detection, and cladding area calculation. Finally, the stainless steel occupancy ratio was obtained.

The method proposed in reference [22] was used to calculate the average cladding thickness. First, the end-face profiles of the specimens were divided into 20 equal parts along the circumference. The cladding thickness corresponding to each part was obtained using a CAD (Autodesk company, San Rafael, CA, USA) measurement tool, and the average of these measurements was calculated.

Electron backscatter diffraction (Carl Zeiss AG, Oberkochen, Germany) was used to observe and count the composite interface grain size, dislocation density, and percentage of recrystallized grains. Here, the scan step was 200 μm. The specific steps are as follows: the composite interface image was first obtained with the help of the electron backscatter diffraction and then imported into Channel5 software for calculating the grain sizes of ferrite and austenite with the grain size statistical function of the software.

A SOPTOP optical microscope and JXA-8230 electron probe (Japan Electronics Co., Akishima-shi, Japan) were used to observe the metallographic organization and distribution of each alloying element in the composite interface, respectively.

A high-temperature Vickers hardness test system (Fuzhen Technology (Beijing) Co., Beijing, China) was used to assess the microhardness of the composite interface; here, the test load was 50 g.

**Figure 6:**
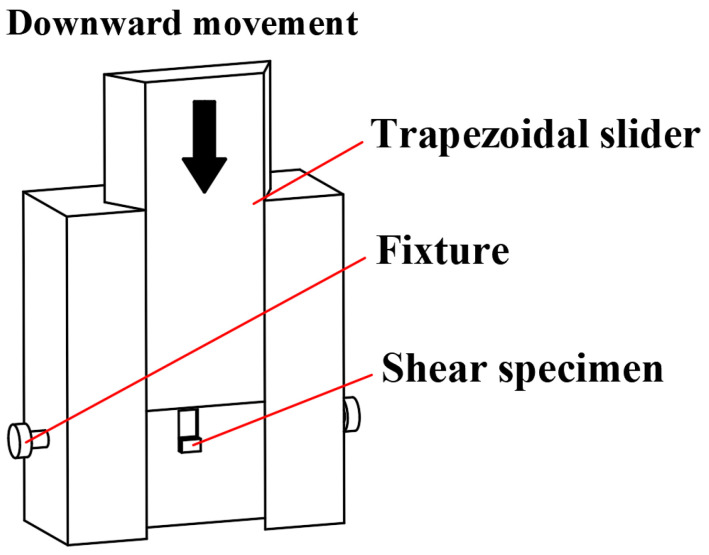
Schematic of the operation of the shear jig (data from Ref. [23]).

**Figure 7:**
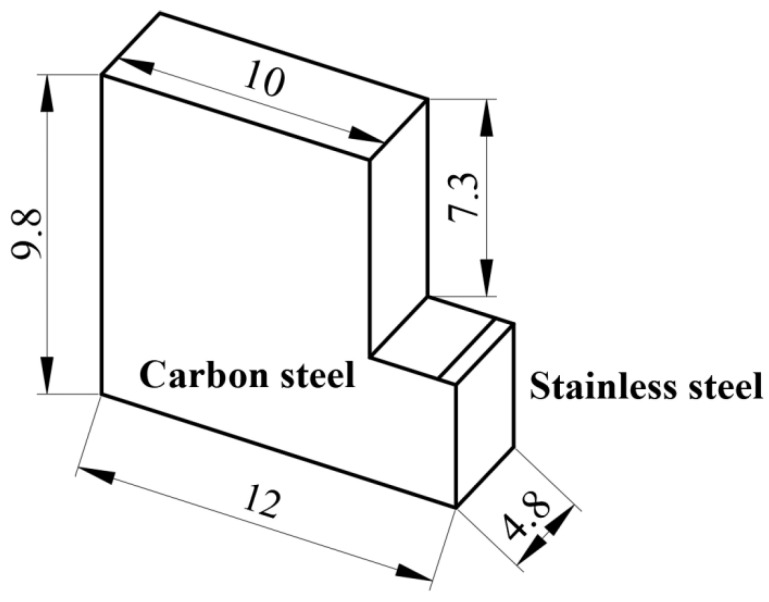
Dimensions of the shear specimens.

The bond strength of the composite interface was tested using the method proposed in reference [23]. Figure 6 shows a schematic of the shearing jig during operation. First, the shear specimen was cut into the shape as shown in Figure 7, then the shear specimen was placed in the shear jig and the cladding of the specimen was sheared from the substrate using a trapezoidal slider. Finally, the bond strength of the composite interface was calculated on the basis of the shearing forces.

**Figure 8:**
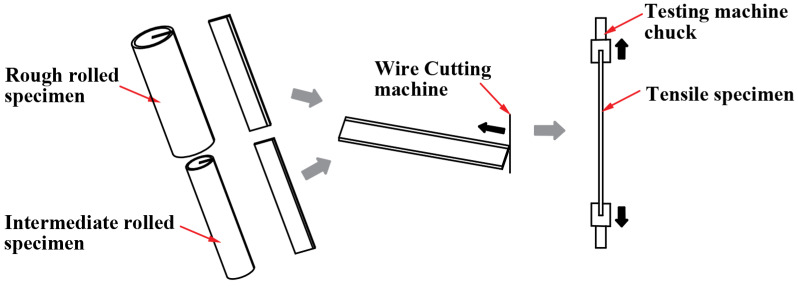
Tensile experiment flow.

**Figure 9:**
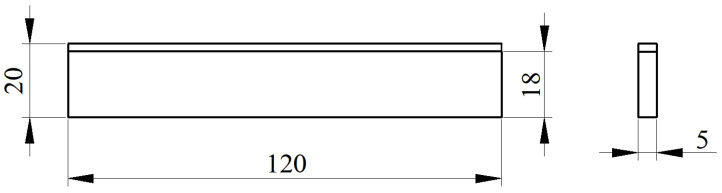
Dimensions of the tensile specimens.

Figure 8 shows a schematic of the tensile test procedure. First, the tensile specimens of specific shapes (as shown in Figure 9) were cut using a wire cutter, and then the electro-hydraulic servo tester (Instron Limited, High Wycombe, UK) was used to test the performance of these tensile specimens.

Figure 8 shows the dimensions of the tensile specimens (unit, mm) and Figure 9 shows a schematic of the tensile test procedure.

The tensile and shear fracture morphologies of the specimens were observed using a scanning electron microscope (Tescan Brno, South Moravia, Czech Republic) at an accelerating voltage of 30 kv.

## 3. Results

### 3.1. Cladding Profile Analysis

**Figure 10:**
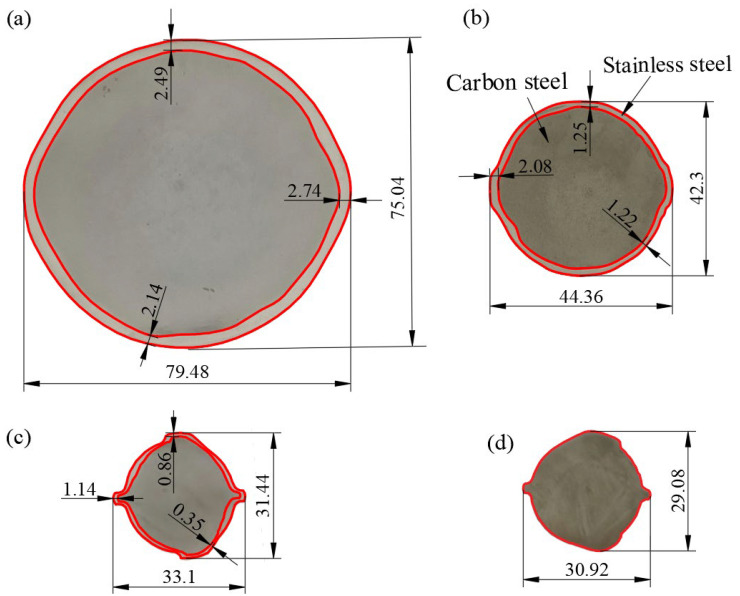
End-face profiles of the specimens: (**a**) Rough-rolled specimen; (**b**) intermediate-rolled specimen; (**c**) clad rebar (data from Ref. [22]); (**d**) HRB400 reinforcement.

Figure 10 shows the end-face profiles of the specimens. The cladding distribution of each specimen was relatively uniform, and no defects, such as dew points, were observed. The minimum cladding thicknesses of the rough- and intermediate-rolled specimens and clad rebars were 2.14, 1.22, and 0.35 mm, respectively; these values are consistently greater than the specified value of 0.18 mm.

Figure 10a,b show the end-face profiles of the rough- and intermediate-rolled specimens, respectively. The formation of “ears,” which is caused by the overfilling of the billet in the hole pattern and the flow of metal toward the roll seam can clearly be observed. Figure 10c shows the end-face profile of the clad rebars. The minimum cladding thickness occurs at the root of the transverse rib owing to the presence of a sharp angle at the bottom of the K1 hole-type crescent groove, causing excessive stress in the cladding stainless steel and thinning of the cladding. Sawicki et al. used Forge2007 software to simulate the fine-rolling of clad rebars and found that the cladding was the thinnest at the root of the transverse rib regardless of the process parameters [24]. Figure 10d shows the end-face profile of an HRB400 rebar rolled under the same parameters. The distance between two longitudinal ribs of the HRB400 bar (30.92 mm) was significantly smaller than that of the clad rebars (33.1 mm), which indicates the greater tendency of the clad rebars to deform along the radial direction during the forming process.

Table 3 shows the average cladding thickness and stainless steel percentage of each specimen. The stainless steel percentage of the specimens fluctuated minimally at approximately 14%. This finding indicates that the cladding stainless steel is deformed at equal proportions during the rolling process. In addition, as the cumulative pressure increased, the average cladding thickness of the specimens decreased from 2.91 to 1.04 mm.

### 3.2. Composite Interface Metallographic Analysis

**Figure 11:**
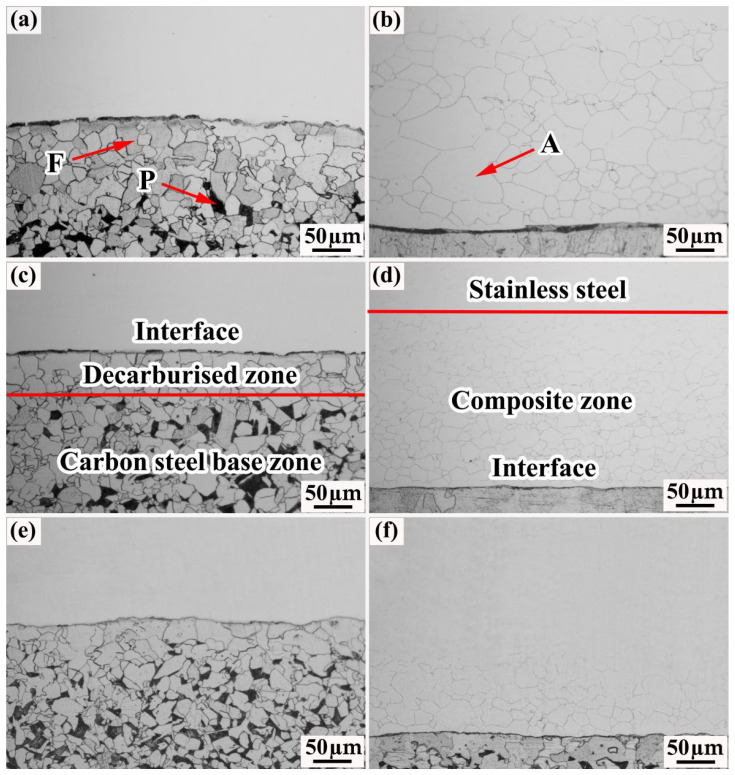
Metallographic images of the specimens: (**a**) Carbon-steel side of the rough-rolled specimen; (**b**) stainless-steel side of the rough-rolled specimen; (**c**) carbon-steel side of the intermediate-rolled specimen; (**d**) stainless-steel side of the intermediate-rolled specimen; (**e**) carbon-steel side of the clad rebar; (**f**) stainless-steel side of the clad rebar.

Figure 11 shows the metallographic images of each specimen. The composite interface of each specimen is relatively straight and no obvious cracks can be observed in the images. Figure 11a,c,e show the metallographic images of the carbon-steel side of each specimen. The carbon-steel side can be divided into two regions, namely, the carbon steel matrix and the decarburization zone. The carbon steel matrix consists of ferrite and pearlite with no bainite. The decarburization zone consists of a single ferrite, which is formed from the diffusion of carbon into the cladding owing to the concentration gradient during the rolling process, which leads to the decarburization of the carbon steel near the composite interface. Closer investigation revealed that the grains in the decarburization zone were significantly larger than those in the carbon-steel matrix, which is due to the ability of pearlite to promote ferrite nucleation during cooling; thus, the grains in the carbon-steel matrix are smaller than those in the decarburization zone [25].

Figure 11b,d,f show the metallographic images of the stainless-steel side of each specimen. Austenite grain areas of varying widths appear in the stainless steel near the composite interface; this area is referred to as the composite zone. The widths of the decarburization and composite zones of the rough-rolled specimens are 87 and 300 μm, respectively, whereas those of the decarburization and composite zones of the clad rebar are only 30 and 110 μm, respectively. These values indicate that the widths of the decarburization and composite zones gradually decrease with an increasing cumulative reduction rate. The above phenomenon can be explained as follows. During the hot-rolling process, the cross-sectional area of the billet extruded by the rolls decreases continuously, which, in turn, decreases the widths of the decarburization and diffusion zones. Wang et al. found that the width of the decarburization layer of a Q235/304 composite plate decreased from 81.82 to 51.51 μm when the cumulative reduction rate was increased from 40% to 70% [26].

### 3.3. Composite Interface EBSD Analysis

**Figure 12:**
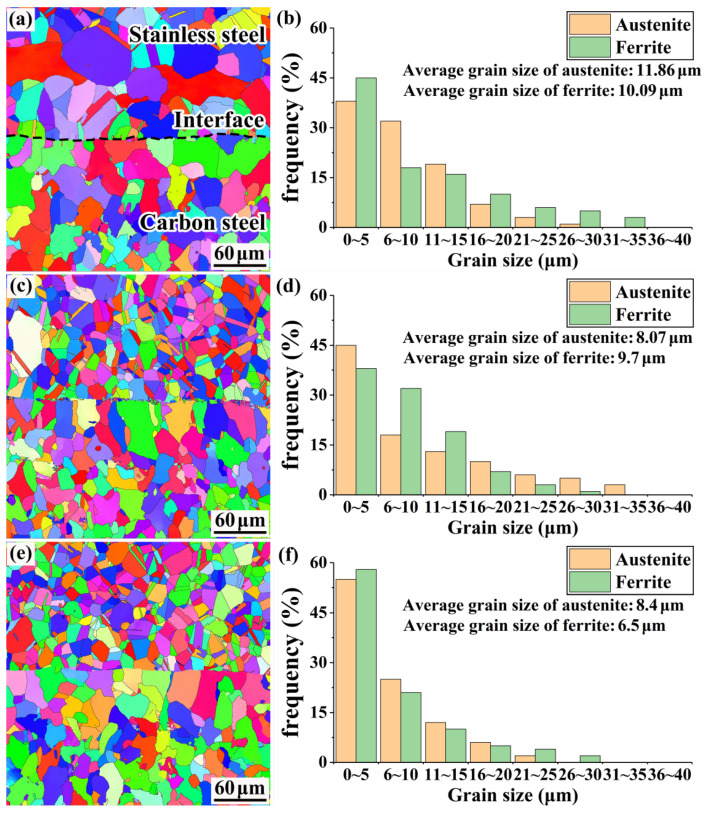
Schematic of the grain size of structures on the composite interface: (**a**) EBSD image of the rough-rolled specimen; (**b**) grain size statistics of the rough-rolled specimen; (**c**) EBSD image of the intermediate-rolled specimen; (**d**) grain size statistics of the intermediate-rolled specimen; (**e**) EBSD image of the clad rebar; (**f**) grain size statistics of the clad rebar.

EBSD was conducted on each specimen to analyze the grain size and recrystallized grain ratio of all structures on the composite interface. Figure 12 shows a schematic of the grain size of various structures on the composite interface. The average grain sizes of ferrite and austenite on the rough-rolled specimens (11.86 and 10.09 μm, respectively) are significantly larger than those on the clad rebars (8.4 and 6.5 μm, respectively), likely because extensive deformation leads to the dynamic recrystallization of the composite interface, resulting in grain refinement. Liu et al. found that the grain size near the interface of an NM300/Q345R composite plate decreases continuously as the compression rate increases and that the pearlite texture changes from rough to fine [27].

**Figure 13:**
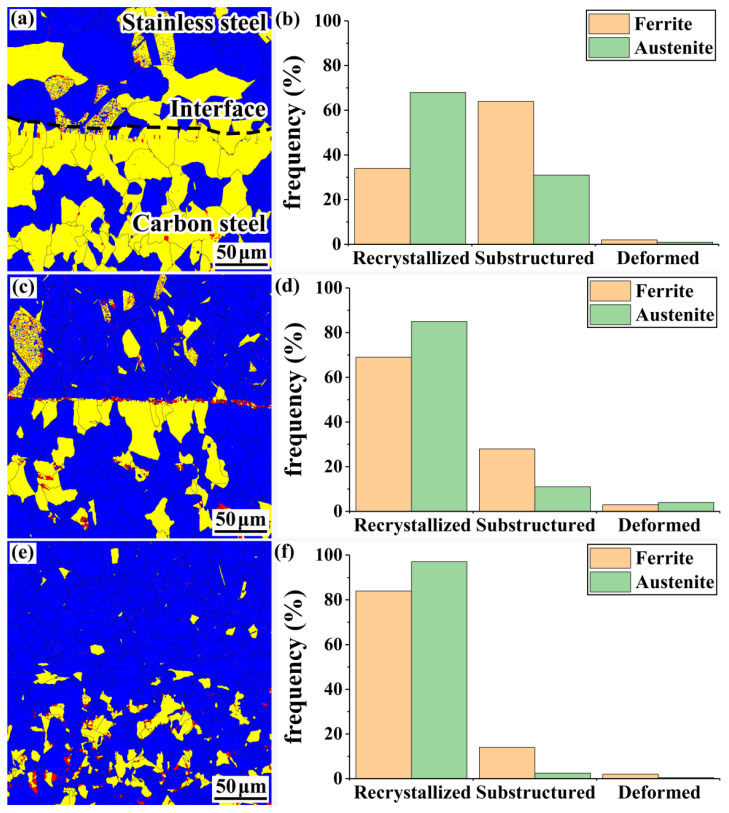
Recrystallization grain ratios of structures on the composite interface: (**a**) DefRex diagram for rough-rolled specimens; (**b**) recrystallization grain statistics for rough-rolled specimens; (**c**) DefRex diagram for intermediate-rolled specimens; (**d**) recrystallization grain statistics for intermediate-rolled specimens; (**e**) DefRex diagram for clad rebars; (**f**) recrystallization grain statistics for clad rebars.

Figure 13 shows the proportion of recrystallized grains in the structures on the composite interfaces. The blue area in the figure represents recrystallized grains, the yellow area represents sub-grains, and the red areas represent deformed grains. Owing to the large difference in properties between the base and cladding materials of the clad rebar, inconsistent deformation is likely to occur during the hot-rolling process of the specimens, leading to dislocation aggregation on the composite interface and an increase in the storage energy of the grains. When the storage energy exceeds a certain value, the grains undergo dynamic recrystallization.

Figure 13a,b show the DefRex plots and recrystallized grain statistics of the composite interface of the rough-rolled specimen, respectively. A large number of sub-grains appear at the carbon-steel side of the rough-rolled specimen, accounting for over 50% of the total number of grains observed. Figure 13c,d depict the DefRex plots and recrystallized grain statistics of the composite interface of the intermediate-rolled specimen, respectively. Compared with the rough-rolled specimen, the number of recrystallized grains in the intermediate-rolled specimen significantly increased; in particular, the percentages of recrystallized grains of ferrite and austenite increased from 34% and 68% to 69% and 84%, respectively. This finding may be attributed to the dynamic recrystallization of the billet, which occurs with each rolling pass. As the number of rolling passes increases, the extent of the dynamic recrystallization of the billet also increases, and the proportion of recrystallized grains in each structure increases accordingly. Figure 13e,f show the DefRex plots and recrystallized grain statistics of the composite side of the clad rebars, respectively. The percentage of recrystallized grains at the stainless-steel side of the clad rebars reaches 97% when the cumulative reduction rate is 82.3%. Interestingly, the proportion of recrystallized grains at the stainless-steel side of each specimen is greater than that at the carbon-steel side, likely because of the higher deformation and internal grain storage energy of the clad stainless steel during the rolling process. A similar law was found by Li et al. in their study on the interfacial structural properties of 06Cr13/Q345R composite plates [28].

In addition, as dynamic recrystallization occurs, the grains re-nucleate and grow at the grain boundaries; thus, the grains are refined as the cumulative reduction rate increases, in agreement with the grain sizes illustrated in Figure 12.

### 3.4. Composite Interface Oxide Distribution and Elemental Diffusion Analysis

**Figure 14:**
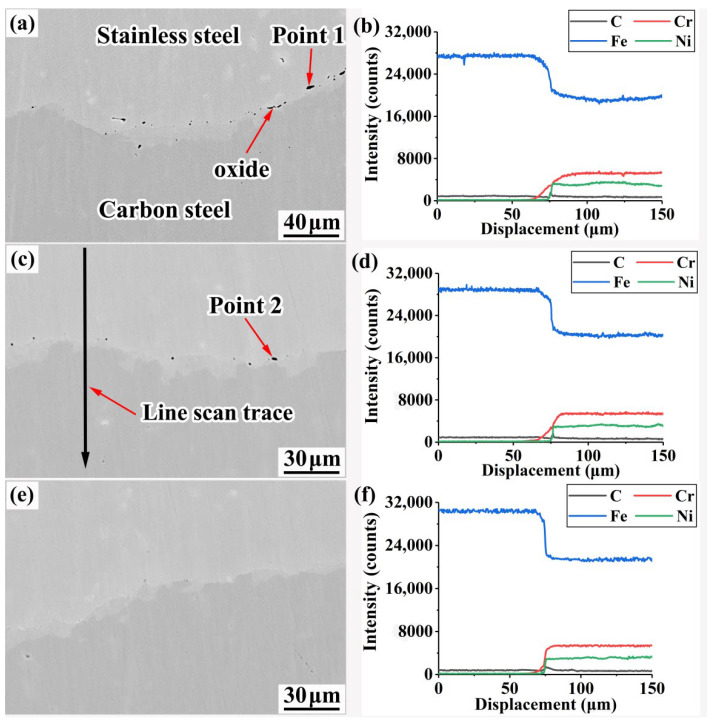
Distribution of elements on the composite interface of the specimens: (**a**) EPMA image of the composite interface of the rough-rolled specimen; (**b**) line scan result of the rough-rolled specimen; (**c**) EPMA image of the composite interface of the intermediate-rolled specimen; (**d**) line scan result of the intermediate-rolled specimen; (**e**) EPMA image of the composite interface of the clad rebar; (**f**) line scan result of the clad rebar.

Figure 14 shows the elemental distribution on the composite interface of the specimens. The diffusion distance of each element in the rough-rolled specimen (C ≈ 65 μm, Fe ≈ 50 μm, Cr ≈ 40 μm, and Ni ≈ 15 μm) is significantly larger than that in the intermediate-rolled specimen, mainly because the diffusion layer is continuously compressed during the rolling process; thus, the diffusion distance of each element decreases accordingly.

As shown by observing the EPMA image of the composite interface of each specimen, a large number of long inclusions appear on the composite interface of the rough-rolled specimen, the largest of which measures up to 10 μm. With the increase of accumulated reduction rate, the number of inclusions on the composite interface is significantly reduced, and the characteristics of these inclusions gradually transform from continuous linear to discontinuous granular. This phenomenon can be explained as follows: (1) As the cumulative compression rate increases, the rolling force on the composite interface (mainly including the pressure perpendicular to the direction of the composite interface and the shear force averaged over the composite interface) increases; thus, the inclusions are increasingly broken. (2) As the rolling process continues, the composite interface area gradually increases, the number of inclusions per unit area decreases, and the composite interface becomes smoother. Zhu et al. found that the inclusions in the HSLA/316L composite plate interface changed from rod-like to granular as the number of rolling passes increased [29]. Surprisingly, fine inclusion was not found in the clad rebar.

Table 4 shows the point sweep results of inclusions in the samples. The main elements in these inclusions are O, Fe, Si, Ca, Mn, and Al; thus, the inclusions may be considered to be oxides. The billets in the present study were vacuumed, but some roughness can still be observed on their surfaces. O can be adsorbed at the pits on the surface of the billets, resulting in incomplete vacuum treatment; thus, during the rolling process, a certain amount of oxides were formed on the composite interface of the specimens.

**Figure 15:**
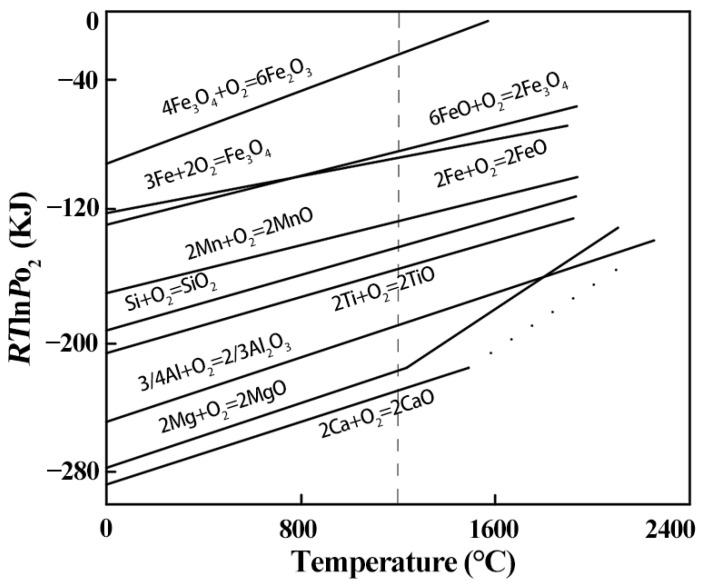
ΔG_0_–Temperature diagram of the metal oxidation reaction.

The order of oxidation of each alloying element depends on the free energy of the generation of the metal (ΔG_0_). The lower the free energy of generation, the more easily the metal is oxidized. Figure 15 shows the ΔG_0_–Temperature diagrams of the metal oxidation reactions. The oxides can be ranked in terms of their free energy of generation (at 1100 °C) as follows: $\Delta G_{\mathrm{CaO}}<\Delta G_{{\mathrm{Al}}_{2}O_{3}}<\Delta G_{{\mathrm{SiO}}_{2}}<\Delta G_{\mathrm{MnO}}<\Delta G_{{\mathrm{Fe}}_{3}O_{4}}$. This ranking shows that, when Fe, Si, Ca, Mn, and Al are also present in the alloy, Ca is oxidized preferentially, followed by Al, Si, Mn, and finally Fe. Since the contents of Ca and Al on the composite interface of the rough- and intermediate-rolled specimens are very low and, thus, insufficient to consume all of the O, the remaining O reacts with Si and Mn, which, in turn, causes the composite interface inclusions to contain Ca, Al, Si, and Mn simultaneously. Nomura et al. found that the main components of the interfacial oxides of stainless steel composite plates under high-vacuum conditions are SiO_2_ and MnSiO_3_, which is attributed to the strong affinity of Si, Mn, and other alloying elements with O [30].

### 3.5. Composite Interface Bond Strength Analysis

Table 5 shows the test results of the bond strength of the composite interface of each specimen. The bond strength of the composite interface of the clad rebar (384 MPa) is significantly greater than that of the rough-rolled (264 MPa) and intermediate-rolled (320 MPa) specimens. This result may be attributed to the fact that the composite interfaces of the rough- and intermediate-rolled specimens contain a certain amount of brittle oxides, which are prone to forming stress concentrations and cause cracks during the shearing process, leading to the premature fracture of the specimens. As the cumulative reduction rate increases, the amount of oxides on the composite interface is continuously reduced, and the dislocations are unable to gather during the deformation process. Thus, the bond strength of the composite interface is continuously improved. The bond strength of the composite interfaces of the above three specimens is notably greater than the value of 210 MPa specified in the Chinese national standard GB/T 6396-2008.

**Figure 16:**
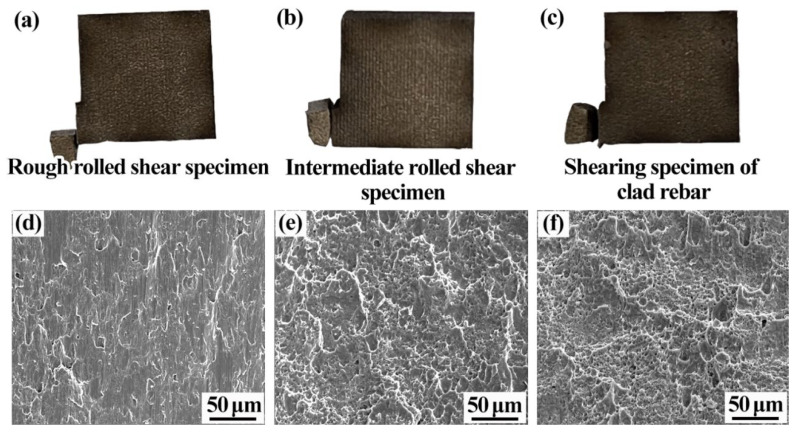
Shear fracture profiles of each specimen: (**a**) Shear fracture diagram of the rough-rolled specimen; (**b**) shear fracture diagram of the intermediate-rolled specimen; (**c**) shear fracture diagram of the clad rebar; (**d**) shear fracture profile of the rough-rolled specimen; (**e**) shear fracture profile of the intermediate-rolled specimen; (**f**) shear fracture profile of the clad rebar.

Figure 16 shows the shear fracture morphology of the specimens. Figure 16a–c show the shear fracture diagrams of the rough- and intermediate-rolled specimens and clad rebar, respectively. Each specimen is fractured at the composite interface, which indicates that the bond strength of the composite interface of each specimen is less than the matrix strength of 20MnSiV and 316L. Figure 16d shows the shear fracture morphology of the rough-rolled specimen, which has a large number of unraveling planes and tearing stripes and is a brittle fracture. Figure 16e shows the shear fracture of the intermediate-rolled specimen. In contrast to the rough-rolled specimen, this specimen shows a certain number of parabolic tough nests at the fracture sites, with a few tearing ribs (mixed tough–brittle fractures). Figure 16f shows the shear fracture morphology of the clad rebar. A large number of small and deep tough nests measuring approximately 4 μm in size appear at the fracture sites of this specimen (ductile fractures). In summary, as the cumulative reduction rate increases, the fracture toughness of the composite interface increases and the fracture mode changes from brittle fracture to ductile fracture. Das et al. conducted tensile–shear tests on composite plates and found that higher numbers of tough nests on the composite interface lead to a higher interfacial bond strength [31].

### 3.6. Tensile Property Test

Owing to the presence of transverse and longitudinal ribs on the surface of the clad rebar, the tensile specimens could not be processed into the specified shapes (as shown in Figure 8). Thus, only the tensile properties of the rough- and intermediate-rolled specimens were tested in this study. The tests were repeated a total of three times.

Table 6 shows the tensile results of the rough- and intermediate-rolled specimens. The average tensile strength of the intermediate-rolled specimens was significantly greater than that of the rough-rolled specimens. This is because the size of each organization’s grains in the intermediate-rolled specimens was smaller, which indicates a larger area of grain boundary in the intermediate-rolled specimens within the same volume. The large number of grain boundaries impeded the movement of dislocations. Therefore, intermediate-rolled specimens feature better tensile properties.

**Figure 17:**
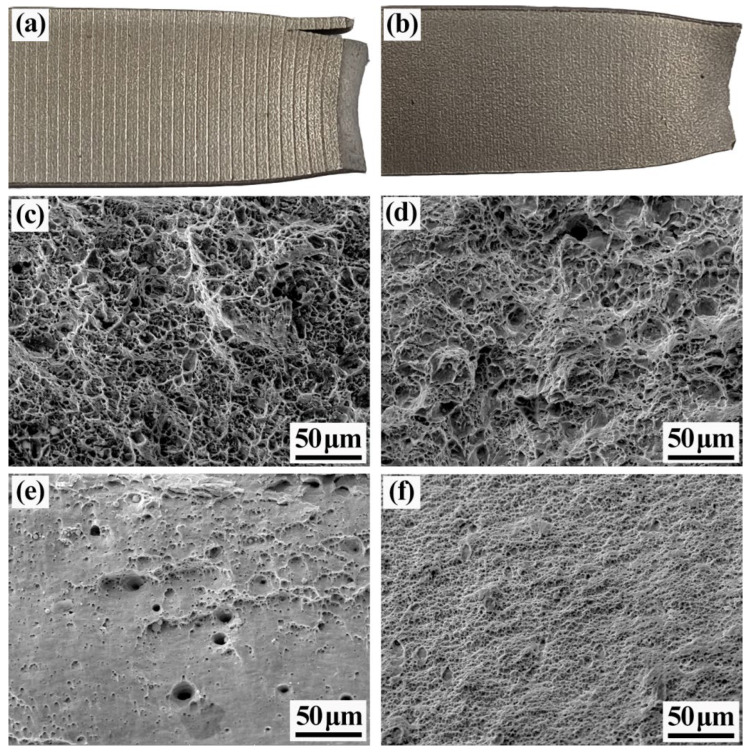
Tensile fracture profiles of rough- and intermediate-rolled specimens: (**a**) Schematic of the tensile fracture of rough-rolled specimens; (**b**) schematic of the tensile fracture of intermediate-rolled specimens; (**c**) tensile fracture profiles of the carbon-steel side of rough-rolled specimens; (**d**) tensile fracture profiles of the carbon-steel side of intermediate-rolled specimens; (**e**) tensile fracture profiles of the stainless-steel side of rough-rolled specimens; (**f**) tensile fracture profiles of the stainless-steel side of intermediate-rolled specimens.

Figure 17 shows the tensile fracture morphologies of the rough- and intermediate-rolled specimens. A remarkable difference between the tensile fracture profiles of the specimens can be observed. Figure 17a shows a schematic of the tensile fracture of the rough-rolled specimen. The composite interface of this specimen is separated. In addition, compared with the stainless steel cladding, the base carbon steel shows more obvious necking, which could be attributed to the smaller deformation, lower hardening, and greater plasticity of the base carbon steel during the rolling process. Figure 17b shows a schematic of the tensile fracture of the intermediate-rolled specimen. After the tensile test, the composite interface of the intermediate-rolled specimen was well bonded without cracks. Liu et al. found that the high bond strength of the composite interface can effectively inhibit the formation and expansion of interfacial delamination cracks and improve the tensile strength and elongation of stainless steel composite plates during the tensile test [32]. Figure 17c shows the tensile fracture morphology of the carbon-steel side of the rough-rolled specimen. A certain number of tough nests and tear ribs appear on the carbon-steel side of the specimen and some of the nests are distributed with secondary-phase particles. Figure 17d shows the tensile fracture morphology of the carbon-steel side of the intermediate-rolled specimen; this side features large and deep tough nests, with no obvious cleavage plane. Figure 17e,f show the tensile fracture morphology of the stainless-steel side of the rough- and intermediate-rolled specimens, respectively. Compared with that of the rough-rolled specimen, the stainless-steel side of the intermediate-rolled specimen has more tough nests and a denser distribution, which indicates that the plastic toughness of the stainless-steel side of the intermediate-rolled specimen is better than that of the rough-rolled specimen.

**Figure 18:**
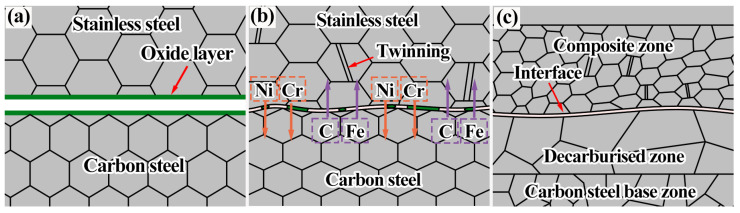
Formation process of the composite interface of the clad rebar: (**a**) Heating state; (**b**) forming process; (**c**) surface structure after rolling.

The present findings can be combined to elucidate the mechanism of composite interface generation of the clad rebar, as shown in Figure 18. Regardless of the atmospheric environment, the billet consistently undergoes oxidation after prolonged heating, forming local oxide films and particles (Figure 18a). These oxides probably include Al_2_O_3_, CaO, and MnSiO_2_. As rolling proceeds, the oxide film gradually breaks up and the fresh metal on both sides of the interface comes into contact with each other under positive pressure to form a solid metallurgical bond. As the cumulative reduction rate further increases, the pores of the composite interface are completely closed. Each alloying element diffuses to the composite interface owing to the concentration difference. Specifically, C and Fe diffuse from the base layer to the cladding layer, whereas Cr and Ni diffuse from the cladding layer to the base layer (Figure 18b). Recrystallization also begins to occur in each structure of the composite interface owing to dislocation aggregation and the grains are continuously refined. After rolling, the interfacial oxides disappear (Figure 18c) and the decarburization and composite zones are formed on both sides of the composite interface.

## 4. Conclusions

1. Compared with HRB400 bars, clad rebars have a greater tendency to deform along the radial direction during rolling; thus, the pass filling degree should be within 0.85–0.9 when designing hole sizes for clad rebars.
2. As the cumulative reduction rate increases, the decarburization zone, width of the composite zone, and diffusion distance of each element gradually decrease. The composite interface oxides change from long strips to granular particles.
3. As the cumulative reduction rate increases, the composite interface dislocations aggregate, grain refinement continues, and the tensile and bonding properties of the specimens are significantly improved.

## Figures and Tables

**Table 1:** Chemical compositions of 20MnSiV and 316L stainless steel (wt%).

Materials	C	Si	Mn	P	S	Cr	Ni	Mo	Fe
20MnSiV	0.21	0.34	1.29	0.007	0.004	0.21	0.03	−	Bal.
316L	0.02	0.71	1.77	0.021	0.007	16.9	12.9	2.25	Bal.

**Table 2:** Rolling Parameters of clad rebar.

Rolling Speed (m/s) in K2 Pass	Rolling Speed (m/s) in K1 Pass	Rolling Mill Type	Roller Material
35.4	48.7	Two-roller rolling mill	55Mn2 alloy forged steel

**Table 3:** Average cladding thickness and stainless steel percentage of each specimen.

Specimen Type	Average Value (mm)	Percentage of Stainless Steel (%)
Rough-rolled specimen	2.91	14.2
Intermediate-rolled specimen	1.59	13.9
Clad rebar	1.04	13.6

**Table 4:** Point sweep results (wt%).

	O	Fe	Si	Ca	Mn	Al
Point 1	30.65	24.39	16.18	4.39	23.61	0.78
Point 2	28.57	39.17	12.52	3.19	15.66	0.89

**Table 5:** Binding strength test results.

Specimen Type	Average Bond Strength (MPa)
Rough-rolled specimen	264
Intermediate-rolled specimen	320
Clad rebar	384

**Table 6:** Tensile test results of the rough- and intermediate-rolled specimens.

Specimen Type	Specimen Number	Yield Strength (MPa)	Tensile Strength (MPa)	Elongation (%)	Average Value (MPa-MPa-%)
Rough-rolled specimens	1	405	584	22	407-588-22.7
	2	408	590	23	
	3	408	592	23	
Intermediate-rolled specimens	1	478	659	32	477.3-658.3-32.6
	2	476	658	33	
	3	478	658	33	
